# A new pterosaur from the early stage of the Jehol biota in China, with a study on the relative thickness of bone walls

**DOI:** 10.1016/j.heliyon.2023.e22370

**Published:** 2023-11-17

**Authors:** Shunxing Jiang, Junyi Song, Xinjun Zhang, Xin Cheng, Xiaolin Wang

**Affiliations:** a Key Laboratory of Vertebrate Evolution and Human Origins, Institute of Vertebrate Paleontology and Paleoanthropology, Chinese Academy of Sciences (CAS), Beijing, 100044, China; b College of Earth and Planetary Sciences, University of Chinese Academy of Sciences, Beijing, 100049, China; c College of Paleontology, Shenyang Normal University, Shenyang, 110034, China; d College of Earth Sciences, Jilin University, Changchun, 130061, China

**Keywords:** *Cratonopterus*, Ctenochasmatidae, Cretaceous, Huajiying formation, Jehol biota, China, Bone wall thickness

## Abstract

The Huajiying Formation (135.4–128.7 Ma) of the northern Hebei represents the early stage of the Early Cretaceous Jehol Biota in China, yielding many kinds of vertebrates. The only known pterosaur specimen was incomplete and assigned to the Ornithocheiroidea. Here we report a more complete pterosaur specimen, assigned to the Ctenochasmatidae. A new taxon is established on two autapomorphies: a large pneumatic foramen present on the ventral surface of the proximal end of the first wing phalanx; and coracoid lacking an expansion at its contact with the scapula, as well as the following combination of characteristics: subsquare sternal plate; coracoid having an extremely concave articulation with a posterior expansion; humerus without a tubercle on the proximal margin between the deltopectoral crest and the head; humerus slightly longer than the wing metacarpal; and the first and third wing phalanges equal in length. The relative thicknesses of bone walls are investigated among pterosaurs in three ways. The overall distribution of R/t ratios shows that most non-pterodactyloids, archaeopterodactyloids, and dsungaripterids have smaller R/t ratios than other groups. Relatively thick bone walls are not unique for the Dsungaripteridae as previously thought, and the humerus and radius of dsungaripterids have thinner walls than other bones. The feature of small R/t ratios is plesiomorphic and the thin-walled humerus and radius of dsungaripterids were evolved to meet the need of the flight, not for frequent take-off and landing as previously thought.

## 1 Introduction

The Early Cretaceous terrestrial Jehol Biota in northeastern China has been famous globally since the early 1990s for producing feathered dinosaurs and many other exceptionally preserved vertebrate fossils [[1], [2], [3]]. This biota was recently divided into three evolving stages, as represented by fossil assemblages from the Huajiying (early), Yixian (middle), and Jiufotang (late) formations [[3], [4], [5]], temporally ranging from the Valanginian to the Aptian (135.4–118.9 Ma) [[6], [7], [8]]. Pterosaurs are well presented in the Jehol Biota, which includes more than 30 genera and species [1,9,10].

The vertebrate-fossil-bearing horizon of the Huajiying Formation (135.4–128.7 Ma), also known as the *Protopteryx* horizon, crops out widely in Fengning County, Hebei Province. The first significant vertebrate fossil from this horizon was the earliest bird in China, *Protopteryx*, discovered at the end of the 20th century [11]. During the last two decades, more vertebrate fossils have been reported from this Lagerstätten, including fishes [5], amphibians [12], birds [13,14], and mammals [5]. The only known pterosaur from the Huajiying Formation was a partial wing and right pes, assigned to the Ornithocheiroidea mainly based on its unusual pedal configuration [15]. It was from the Senjitu Basin, where the deposits are slightly younger than the same formation in the Sichakou Basin [15]. Except for this incomplete specimen, the earliest records are still from the base of the Yixian Formation (125.6 Ma) [9,16,17], the middle stage of this biota [5]. Here, we provide a description of another more complete pterosaur specimen from this horizon, which is assigned to the Ctenochasmatidae. Meanwhile, we study the relative bone wall thickness in pterosaurs, especially providing more information about the Dsungaripteridae.

## 2 Materials and methods

### 2.1 Materials

The holotype of *Cratonopterus huabei* gen. et sp. nov. (IVPP V 14935) was collected nearly two decades ago from Fengning County, Hebei Province. Material for the study of the relative thickness of the bone walls is listed in Table S1. Among these taxa, the cortical thickness of *Dsungaripterus*, *Noripterus*, *Seripterus*, *Kryptodraco*, *Gegepterus*, *Sinopterus*, *Huanhepterus*, and an indeterminate ctenochasmatid are based on our measurements, and the others are derived from the literature, especially the doctoral thesis of Martin-Silverstone [18].

## 3 Methods

*Cratonopterus huabei* gen. et sp. nov. was examined under a Zeiss Stemi 508 microscope, and the largest two pieces were scanned by nano-CT. One histological thin section was made from the mid-shaft of the second wing phalanx.

To obtain the relative thickness of bone walls, three methods were employed. (1) Broken bones were directly measured with callipers (represented by CAL in Table S1). The bone wall thickness (t) and the diameter (D) of the bone were measured, and the R/t ratio is half of the D/t ratio. Because only thicknesses at the original breakages can be measured by callipers, the exact positions of the R/t ratios are labelled. (2) Bones with histological thin sections (represented by HST in Table S1). Besides the published thin sections, additional thin sections were made, and new images were taken using an HD digit camera on the polarized light microscope SDPTOP CX40P. Measurements were determined in Adobe Photoshop. (3) Bones with CT scanning (represented by CT in Table S1). Some long bones were scanned by nano-CT, and adjusted images were obtained in VGStudio Max 3.0. These measurements were also obtained using Adobe Photoshop. Because the cross-sections of the bones are irregular, and the medullary cavity is elliptical or subelliptical, more regular than the cortex, the centres of the medullary cavity were considered the centres for the whole cross-sections. The ratios in different directions were calculated, and at least the directions with the thinnest or thickest bone walls were included if possible. The mean R/t ratio represents the relative thickness for one cross-section.

All experiments were performed in the Key Laboratory of Vertebrate Evolution and Human Origins, IVPP.

## 4 Results

### 4.1 Systematic palaeontology

Order PTEROSAURIA Kaup 1834 [19].

Suborder PTERODACTYLOIDEA Plieninger 1901 [20], Family Ctenochasmatidae Nopcsa 1928 [21] sensu Andres 2014 [22].

*Cratonopterus* gen. nov.

*Derivation of name.* “Craton”, from the Greek “Kratos”, means a stable part of the Earth's continental crust, referring to the region where the specimen was found and also in honour of the first Basic Science Center Project of the National Natural Science Foundation of China in Earth Science *Craton destruction and terrestrial life evolution*, and “pterus”, in Greek, means “wing”, often used for pterosaurs.

Type species. *Cratonopterus huabei* sp. nov. by monotype; Diagnosis. As for type species.

*Cratonopterus huabei* sp. nov.

**Fig. 1:**
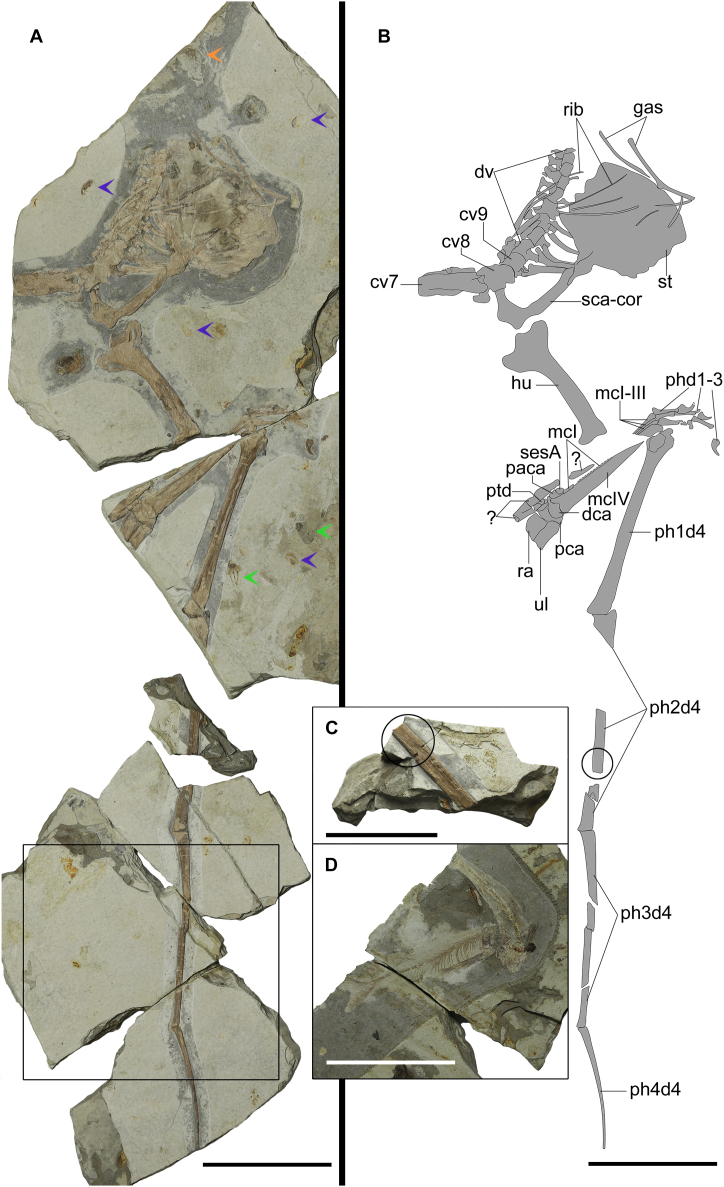
Photos and line drawing of *Cratonopterus huabei* gen. et sp. nov., IVPP V 14395. A, the photo of the whole skeleton. Violet, green, and orange arrows indicate associated conchostracans, mayfly larvae, and a fish fin respectively on the same surface. B, line drawings of the whole skeleton. C, close-up of the smallest slab before the histological sample (in the circle) was collected. D, two fish on the opposite surface of the pieces with wing phalanges (in the frame of A). Abbreviations: cv7-9, seventh to ninth cervical vertebrae; dca, distal syncarpal; dv, dorsal vertebrae; gas, gastralia; hu, humerus; mcI-IV, metacarpals I-IV; paca, preaxial carpal; pca, proximal syncarpal; phd1-3, manual digits I-III; ph1-4d4, first to fourth phalanges of manual digit IV; ptd, pteroid; ra, radius; ri, rib; sca-cor, scapulocoracoid; sesA, sesamoid A; st, sternum; ul, ulna; ?, uncertain. Scale bars, 100 mm in A, B, and D, and 50 mm in C.

**Fig. 2:**
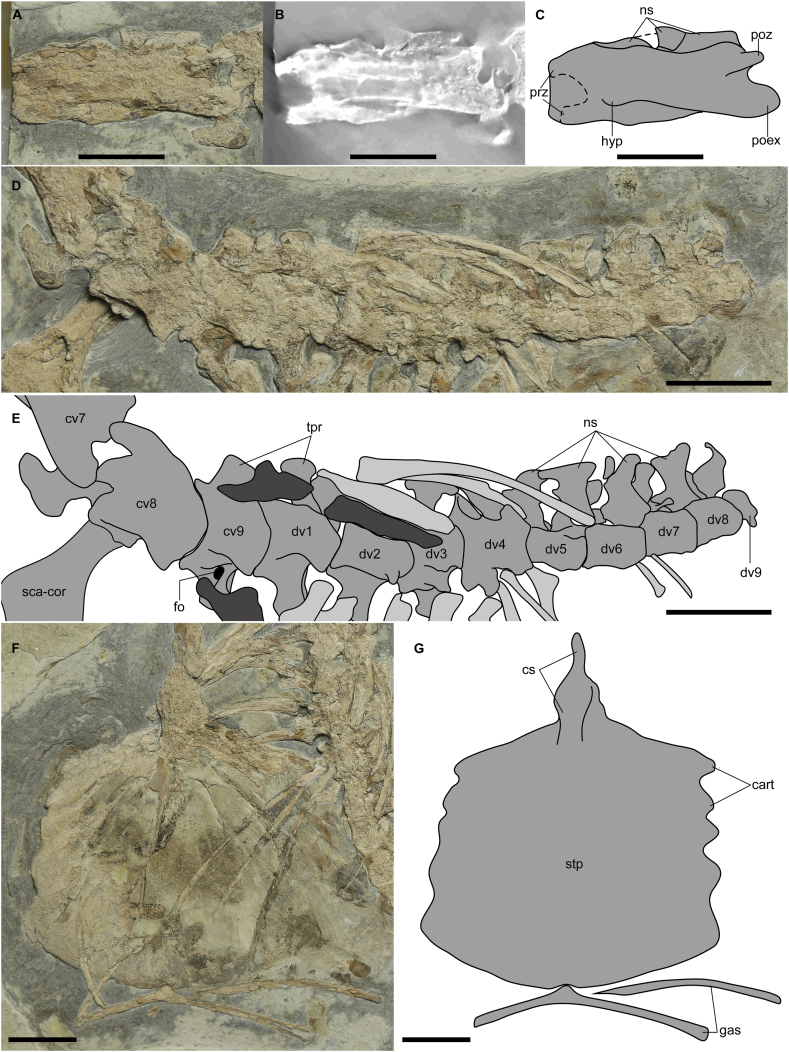
Close-up of the axial skeleton of *Cratonopterus huabei* gen. et sp. nov. (IVPP V 14395). A, B, and C, the seventh cervical vertebra. D and E, other vertebrae. F and G, sternum and gastralia. A, D, and F, photos. B, CT image. C and E, line drawings. Cervical and dorsal ribs are indicated in dark and light grey, respectively. Abbreviations: cart, costal articulation; cs, cristospine; cv7-9, seventh to ninth cervical vertebrae; dv1-9, first to ninth dorsal vertebrae; fo, foramen; gas, gastralia; hyp, hypapophysis; ns, neural spine; poex, postexapophysis; poz, postzygapophysis; prz, prezygapophysis; sca-cor, scapulocoracoid; stp, sternal plate; tpr, transverse process. Scale bars, 20 mm.

**Fig. 3:**
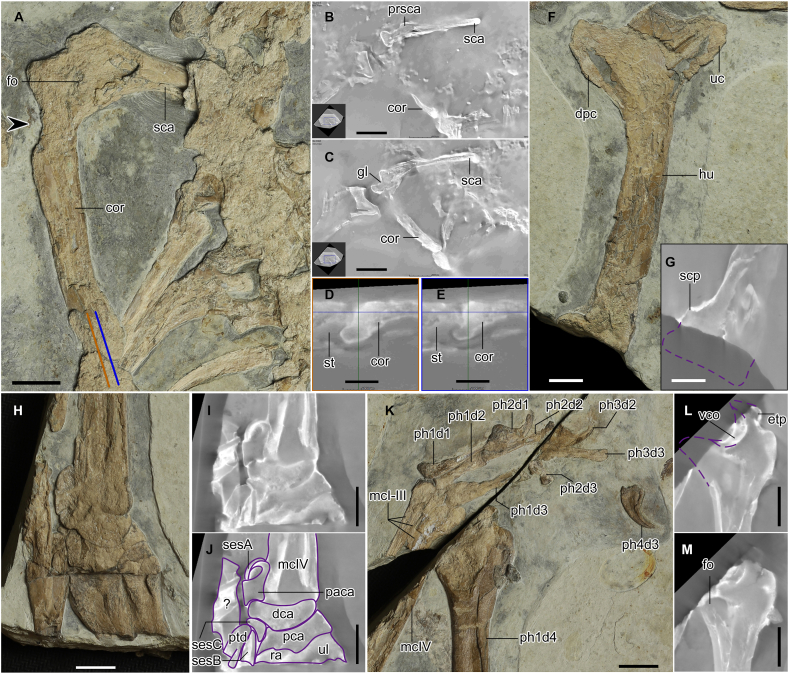
Close-up of the pectoral girdle and the forelimb of *Cratonopterus huabei* gen. et sp. nov. (IVPP V 14395). A-E, scapulocoracoid. F and G, humerus. H-J, carpal region. K-M, manual phalanges. A, F, H, and K, photos. B-E, G, I, L, and M, CT images. J, line drawings. Orange and blue lines in A indicate the positions of CT images shown in D and E, respectively. The arrow indicates the position without the expansion of the coracoid. Abbreviations: cor, coracoid; dca, distal syncarpal; dpc, deltopectoral crest; etp, extensor tendon process; fo, foramen; gl, glenoid fossa; hu, humerus; mcI-IV, metacarpals I-IV; paca, preaxial carpal; pca, proximal syncarpal; ph1-2d1, first to second phalanges of manual digit I; ph1-3d2, first to third phalanges of manual digit II; ph1-4d3, first to fourth phalanges of manual digit III; ph1d4, first phalanx of manual digit IV; prsca, scapular process; ptd, pteroid; ra, radius; sca, scapula; scp, supracondylar process; sesA-C, sesamoids A-C; st, sternum; uc, ulnar crest; ul, ulna; vco, ventral cotyle; ?, uncertain. Scale bars, 10 mm in A, F, G, and H-M, 20 mm in B and C, and 0.5 mm in D and E.

**Fig. 4:**
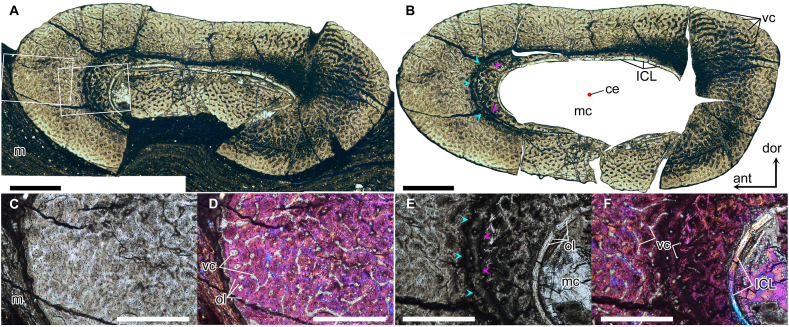
Histological thin section of the second wing phalanx of *Cratonopterus huabei* gen. et sp. nov. (IVPP V 14395). A, composite photo of the whole thin section. B, reconstruction of the cross-section. C and D, close-up of the left frame in A. E and F, close-up of the right frame in A. A-C and E, under the plane polarized light; D and F, under the crossed polarized light with a gypsum plate (one λ). The blue and pink arrows indicate two lines of arrested growth (LAGs), respectively. Abbreviations: ant, anterior; ce, the centre of the cross-section; dor, dorsal; m, matrix; mc, medullary cavity; ICL, internal circumferential layer; ol, osteocyte lacuna; vc, vascular canal. Scale bars, 1 mm in A and B, and 0.5 mm in C–F.

Fig. 1, Fig. 2, Fig. 3, Fig. 4

*Derivation of name.* “huabei”, in Chinese pinyin, means “north China”, referring to the North China Craton.

*Holotype.* Partial skeleton, including vertebrae (cervical and dorsal), the sternum, the right scapulocoracoid, and most elements of the right wing, housed at the Institute of Vertebrate Paleontology and Paleoanthropology, Chinese Academy of Sciences (IVPP V 14395), Beijing, China (Fig. 1, Fig. 2, Fig. 3, Fig. 4).

*Diagnosis*. *Cratonopterus huabei* is a medium-sized archaeopterodactyloid, which can be distinguished from all other members of the clade by the following autapomorphies: a large pneumatic foramen present on the ventral surface of the proximal end of the first wing phalanx; and coracoid lacking an expansion at its contact with the scapula. The specimen can be further distinguished from other archaeopterodactyloids on the basis of the following combination of characteristics: subsquare sternal plate; coracoid having an extremely concave articulation with a posterior expansion; humerus without a tubercle on the proximal margin the deltopectoral crest and the head; humerus slightly longer than the wing metacarpal; and the first and third wing phalanges equal in length.

*Distribution*. Fengning, Hebei Province, China; Huajiying Formation, Early Cretaceous (middle Valanginian to middle Hauterivian) [6].

### 4.2 Description of *Cratonopterus huabei* gen. et sp. nov

*Generalities*. The only known specimen of *Cratonopterus huabei* was found in beige-coloured shales and was brought to IVPP in six pieces (Fig. 1). The largest two pieces, containing most of the skeleton, could be united based on a perfect match of rocks and bones. Another three pieces contained wing phalanges and could also be pieced together. The last and the smallest piece with part of the second wing phalanx did not show any contact surface with the other material. However, based on the colour of the matrix and the preservation of the bones, it is reasonable to assign them all to one individual. The total length of the preserved forelimbs (humerus, wing metacarpal, and the first, third, and fourth wing phalanges) is 0.63 m, indicating an estimated wingspan of approximately 1.8 m. Other fossils associated with this pterosaur on the same surface include a fish fin and large numbers of conchostracans and mayfly larvae (Fig. 1A and B). Two nearly complete fish are preserved on the opposite surface of the pieces with wing phalanges (Fig. 1D).

*Vertebrae*. The last three cervical vertebrae, the seventh to ninth, are preserved. The seventh cervical is exposed in lateroventral view (Figs. 1, 2A-C). CT imaging reveals that both prezygapophyses are preserved. This cervical has a length of 52.65 mm between pre- and postzygapophyses and a width of 15.12 mm between two prezygapophyses, which is slightly larger than the width in the middle. Hence, a length/width ratio of mid-cervicals is at least 3.5, indicating extremely elongated mid-cervical vertebrae, typical for the Archaeopterodactyloidea and Azhdarchoidea [23,24]. The postexapophysis is present, and the hypapophysis is discernible in the CT image. The neural spine forms a low ridge. No foramina were seen. The eighth cervical is exposed in lateral view, and it is much shorter than the seventh. The last cervical is even shorter than the eighth one but has a similar structure to the first dorsal vertebra in ventral view and bears a pair of transverse processes. The right process has a foramen at its base and still contacts the cervical ribs.

There are nine dorsal vertebrae preserved. The first four are exposed in ventral view, the fifth to eighth in lateral view (Fig. 2D and E). A small fragment next to the eighth dorsal is interpreted as the ninth one. No notarium is present in this specimen because of the separation between the first and second dorsal vertebrae, and no supraneural plate can be detected in CT images. The absence of the notarium is the major condition except for the Dsungaripteromorpha and Pteranodontoidea [24], different names including similar taxa in different clades in some other phylogenies, such as [25].

*Sternum*. The sternum is well preserved in ventral view (Fig. 2F and G). The length (105.77 mm) is longer than the width (88.86 mm of posterior margins). The cristospine accounts for 25.8 % of the sternal length, smaller than the ratio in *Forfexopterus* and *Elanodactylus* [26,27]. The anterior portion of the cristospine is much thinner than its posterior portion. The articular facets for coracoids on the cristospine cannot be detected. The sternal plate is broad, thin, and subsquare. It has a straight anterior margin and a slightly curved posterior margin with the former shorter than the latter. The lateral margins have a scalloped appearance, including five costal articulations along each margin. The sternal keel is weak, extending slightly beyond the anterior margins.

*Ribs*. The cervical ribs are stout and double-headed, similar to the morphology, but shorter than that of the first pairs of dorsal ribs (Fig. 1, Fig. 2; 40.12 mm *versus* 49.65 mm). The posterior ribs are curved and slender, and whether they bear double heads is unknown. From the fifth dorsal vertebra, no more ribs still contact the dorsal transverse processes.

*Gastralia*. The first gastralium is thin and curved, and no unfused segments can be distinguished (Fig. 2). In the midline, there is an anterior projection, which still articulates with the notch of the posterior margin of the sternum. The two lateral halves form an angle of approximately 150°.

*Scapulocoracoid*. The coracoid and the distal part of the scapula are exposed in posterior view, and the rest of the scapula remains in the matrix (Fig. 3A–C). The scapula and coracoid are fully fused, and the former is slightly longer than the latter (69.10 *versus* 64.42 mm). The scapula is straight, and the coracoid is gently curved. The proximal end of the coracoid has a strongly concave articulation with a posterior expansion (Fig. 3D and E). The coracoid does not have an expansion at its contact with the scapula, distinguishing it from other archaeopterodactyloids, such as *Pterodactylus*, *Gegepterus*, *Elanodactylus*, and *Forfexopterus* [26,[28], [29], [30]]. The coracoid process (or biceps tubercle) is less prominent than other pterosaurs, such as *Kunpengopterus* [31], *Hamipterus* [32], *Anhanguera* [33], and *Dsungaripterus* [34], but taphonomic crushing can result in the partial absence of this process. Two pneumatic foramina are present. One lies behind the glenoid fossa, which is also reported in *Pteranodon* and *Noripterus* [34,35], but the size is much smaller in the new material. The other is on the coracoid, at the corner of the coracoid process and the ventral margin of the glenoid fossa, which is common in many pterosaurs, such as wukongopterids, *Anhanguera*, *Dsungaripterus*, *Noripterus*, *Hamipterus*, and *Quetzalcoatlus* [[32], [33], [34],^36^,^37^].

*Humerus*. The right humerus is slightly curved in anterior view (Fig. 3F). The distal-most portion is missing, and the proximal part of the supracondylar process can be observed in the CT images (Fig. 3G). Based on this process, the distal part of the humerus is reconstructed with an estimated length of approximately 113 mm. The deltopectoral crest is relatively short and proximally located, which is similar to that of other archaeopterodactyloids, such as *Pterodactylus* [28], *Huanhepterus* [38], and *Forfexopterus* [26]. It is worth noting that this crest is also similar to that of *Noripterus complicidens* [34] and that the exact condition of *Dsungaripterus* is unknown. No foramina can be detected in the new material.

*Radius and ulna*. Only the proximal ends of the right radius and ulna were preserved (Fig. 3H–J). The ulna is overlapped by the radius.

*Carpals and pteroid*. The right proximal and distal syncarpals are exposed in dorsal view, although the surfaces are missing (Fig. 3H–J). The preaxial carpal lies next to the proximal end of the wing metacarpal, and sesamoid A is a roundish element overlapping the preaxial carpal. Sesamoid B is a short stick-like bone, with proximodistal elongation. Sesamoid C is triangular, and it lies at the corner between the proximal and distal syncarpals and contacts the pteroid. The pteroid only has its proximal part preserved.

*Metacarpal*. The wing metacarpal is incomplete, but the whole length (102.81 mm) can be confidently estimated due to the positions of both ends (Fig. 1). The wing metacarpal is much stronger than the others, a common condition in the Pterosauria. At the proximal end, only a short part of metacarpal I can be observed next to the wing metacarpal (Fig. 3H–J). Along the shaft of the wing metacarpal, the impression of a slender bone is interpreted as metacarpal I. Hence, it is certain that at least metacarpals I and IV contacted the distal syncarpal. Metacarpals I-III can be observed at the distal end, and they are nearly articulated with the three digits (Fig. 3K).

*Digits I-III*. The first digit is the smallest, and the third is the longest (Fig. 3K). The first phalanges of the first and third digits are longer than the other phalanges. The second phalanx of digit III is extremely short, and the first phalanx of digit III has an abductor tubercle, similar to *Noripterus* [34], *Pteranodon*, and *Pterodactylus* [35], which is probably common on broad taxonomic scales. The unguals are large and have a groove in the middle of the distal proportion.

*Wing phalanges*. The first wing phalanx is almost complete and exposed in dorsal view (Fig. 1). The extensor tendon process is partially preserved, and it fuses with the shaft of the first wing phalanx (Fig. 3L). A large foramen is present beneath a break at the proximal end on the dorsal surface (Fig. 3M), which was reported in *Pteranodon*, *Nyctosaurus*, and *Anhanguera* but absent in non-pterodactyloids, other archaeopterodactyloids, *Noripterus*, and *Dsungaripterus* [24]. The second wing phalanx is incomplete, and the third and fourth wing phalanges are nearly complete. The fourth wing phalanx is slightly curved. The lengths of the first and third phalanges are similar, and the fourth is about two-thirds of their lengths.

*Histological results*. The thin section is taken from the mid-shaft of the second wing phalanx, probably closer to the distal end rather than the proximal end (Fig. 1C). The bone wall is between 1.02 and 2.06 mm thick, and the anterior and posterior parts are the thickest (Fig. 4A). Based on the reconstruction, the mean R/t ratio is approximately 1.78 (Fig. 4B), which is in the range of dsungaripterid pterosaurs [39,40]. The cortex is composed of primary tissue, with numerous longitudinal or reticular vascular canals (Fig. 4C–F), indicating fast growth before death. The vascular canals and cracks in the section were filled with calcite. Two close incomplete lines of arrested growth (LAGs) are preserved in the anterior region, and the rest of them were absorbed by the expansion of the medullary cavity. The internal circumferential layer (ICL) with elongated osteocyte lacunae is located at the endosteal surface. It is thin and avascular, and its extinction occurs in different regions under the crossed polarized light. The presence of the ICL indicates that the expansion of the medullary cavity ceased. The bone tissue in the posterior region shows a very dark colour, which might be caused by bacterial infections [41].

### 4.3 The relative thickness of bone walls

**Fig. 5:**
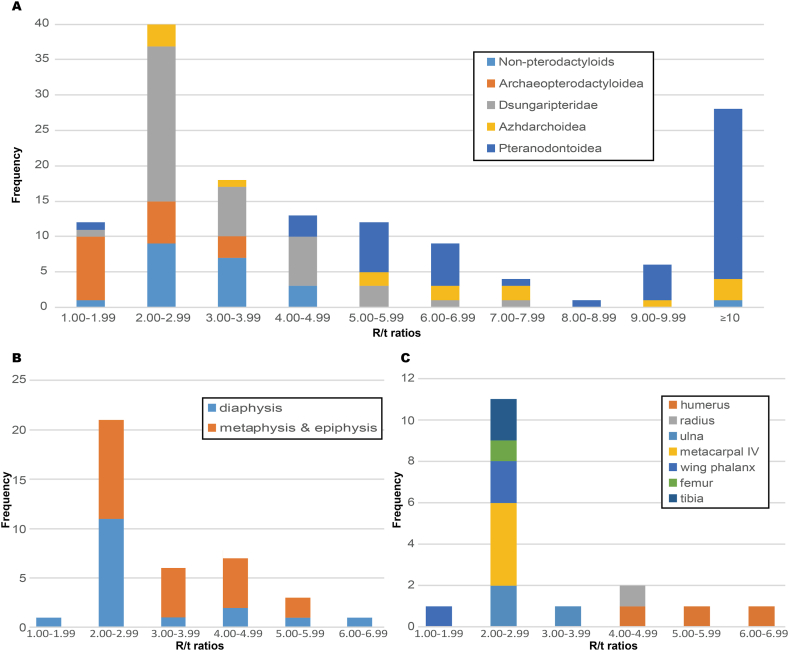
Histograms of the R/t ratios in samples of pterosaurs, based on the data in Table S1. A, frequency of R/t ratios in samples of different pterosaur groups; B, frequency of R/t ratios in samples from different positions of a long bone in dsungaripterids; C. frequency of R/t ratios in samples from the diaphyses of different bones in dsungaripterids.

The R/t ratios, representing the relative thickness of bone walls, vary among the Pterosauria. Based on our dataset, the non-pterodactyloids, archaeopterodactyloids, and dsungaripterids mostly have smaller R/t ratios than those of azhdarchoids and pteranodontoids (Fig. 5A, Table S1), comfirming the previous result that relative bone wall thicknesses are phylogenetic related, and that bone walls of non-pterodactyloids and primitive pterodactyloids are relatively thick [18,40]. Our investigation about dsungaripterids reveals that not all wing bones of dsungaripteroids have low R/t ratios as previously thought (Fig. 5B and C, Table S1) and that many factors can influence the R/t ratios of pterosaurs.

## 5 Discussion

### 5.1 The ontogeny of the holotype of *Cratonopterus huabei* gen. et sp. nov

It is crucial to determine the ontogenetic stage of a particular specimen in many pterosaur studies. Body size can sometimes be used to determine the ontogenetic stage, but it is not a good criterion [42]. Size-independent criteria used for pterosaurs include morphological criteria, such as the degree of ossification, the fusion of particular elements [28,33,42,43], and histological criteria [[44], [45], [46], [47]].

The holotype of *Cratonopterus huabei* is considered an adult based on fusions of the proximal syncarpal, distal syncarpal, scapulocoracoid, and the first wing phalanx and its extensor tendon process based on Kellner's [43] catalogue. However, this result is inconsistent with the histological phenomena. The absence of the external fundamental system (EFS) and numerous vascular canals in the periosteal region of the cortex reveals that the growth speed of the second wing phalanx was still fast before death [44,47]. This indicates that all the expected fusions occurred before the cessation of bone growth in this individual, which is contrary to the sequence in *Tropeognathus* cf. *mesembrinus* [48]. On the basis of the existing data, inconsistencies in ontogenetic stages based on morphological and histological criteria are present among pterosaurs. The limited discoveries of EFS and limited knowledge about the termination of bone growth [44,[47], [48], [49], [50]] confuse the inconsistency. Another possible interpretation is that Kellner's fusion sequence is not valid for all pterosaurs because of different tempos of skeleton development [51]. No matter which interpretation is adopted here, the new specimen is a growing subadult individual, probably close to the adult stage.

### 5.2 The bone wall thickness of pterosaurs

Unwin et al. [39] and Unwin [52] proposed that relatively thick bone walls are characteristics of dsungaripteroids and possibly unique to this clade. R/t ratios were used to describe the radius (R) of the shaft compared with the bone wall thickness (t). The R/t ratios of typical pterosaur wing bones are between 3.0 and 10.0 [39]. Fastnacht [40] agreed that the dsungaripteroids had thicker bone walls than the other clades. The dsungaripterids had R/t ratios from 1.6 to 2.1, while other pterosaurs had ratios from 7.0 to 20 [40]. This characteristic was also accepted by other researchers only with slight differences in the range of the ratios among dsungaripteroids [[53], [54], [55]]. Meanwhile, Fastnacht [40] also found that non-pterodactyloids and primitive non-dsungaripteroid pterodactyloids had thicker bone walls than other pterodactyloids but still significantly thinner than in dsungaripteroids. Martin-Silverstone [18] presented a large dataset of wing bones, revealing that bone walls of dsungaripteroids, non-pterodactyloids, and ctenochasmatids are relatively thicker than other groups.

To investigate the relative thickness of the bone walls among pterosaurs, 143 R/t ratios were studied from the literature or our new measurements, including more than 30 taxa (see Table S1). The overall distribution of R/t ratios shows that most non-pterodactyloids, archaeopterodactyloids and dsungaripterids have smaller R/t ratios than other groups, displaying a similar result to that of Martin-Silverstone [56]. Because about half of the R/t ratios are derived from Martin-Silverstone [56], the result is expected. According to recent phylogenetic analyses [24,25], non-pterodactyloids and archaeopterodactyloids lay at the base of the Pterosauria. Limited information on possible pterosaurian ancestors revealed relatively thick bone walls, with R/t ratios of 2.28 in aphanosaurian *Teleocrater* [57] and 2.50–3.57 in lagerpetid *Dromomeron* [58,59]. Hence, this feature is most likely plesiomorphic to pterosaurs based on parsimony. Then, dsungaripterids inherit this feature from their ancestors, and relatively thin bone walls evolved multiple times in the pterosaurian evolutionary history.

Compared with previous studies on bone wall thickness, we provide a comprehensive dataset of dsungaripterids. More than half the R/t ratios of dsungaripterids are small (<3), but the others are larger, different from the previous ranges for this clade [39,40]. Our result shows a variation in the thickness from the different positions of a long bone (Fig. 5B). More complete limb bones of *Dsungaripterus* and *Noripterus* were CT scanned, and the result confirms that the bone walls of the mid-shaft are thickest (Fig. S1). The histological thin sections of the ulna and the fourth wing phalanx ([60], Figs. 2.46-2.48) and the CT scanning of three first wing phalanges [61] obtained a similar phenomenon. This is the main reason for the large R/t ratios found in the Dsungaripteridae. Generally, the mid-shaft is considered the part with the most deposition of limb bones [47], and this probably leads to the thickest wall.

The R/t ratios also vary among different limb bones. Among the R/t ratios from the mid-shaft of dsungaripterids in our dataset, humeri and a radius have larger values than others (>4.7, Fig. 5C, Table S1). Hence, there is no necessary connection between the relatively thick bone walls and dsungaripterids. This pattern occurs also in two specimens of *Rhamphorhynchus* (RAM 14522 and SMNS 9620) and some pteranodontoids, but is inconsistent with some other specimens, such as *Germanodactylus rhamphastinus* (BSPG 1977.XIX.1) and *Anhanguera* (AMNH FARB 22552), indicating this pattern is not plausible for all pterosaurs. However, it is worth noting that the same pattern was discovered in bats, whose humeri and radii have the smallest wall thickness in limb bones [62]. The higher the R/t-ratio of a hollow tube-like bone, the more likely local buckling of the structure will be due to the compressive force in the direction of the longitudinal axis, but the more torsional and bending strength the bone will obtain [40,63]. The humerus and radius in bats are subjected to large torsional loads during flight, the need to resist loads of torsion may drive the bones to reduce the cortical thickness [62]. In birds, the humerus, ulna and femur generally process torsion-resisting features due to the same need [64]. Fastnacht [40] proposed that relatively thick bone walls in dsungaripterids are related to frequent take-off and landing. Because of the discovery of the thin-walled humerus and radius, the same pattern as bats, it is reasonable to speculate the possibility of the frequent take-off and landing, which is not common in bats. The preferred interpretation here is that thick bone walls in dsungaripterids are plesiomorphic as mentioned above, and humeri and radii evolved thin-walled to meet the needs of the flight.

**Fig. 6:**
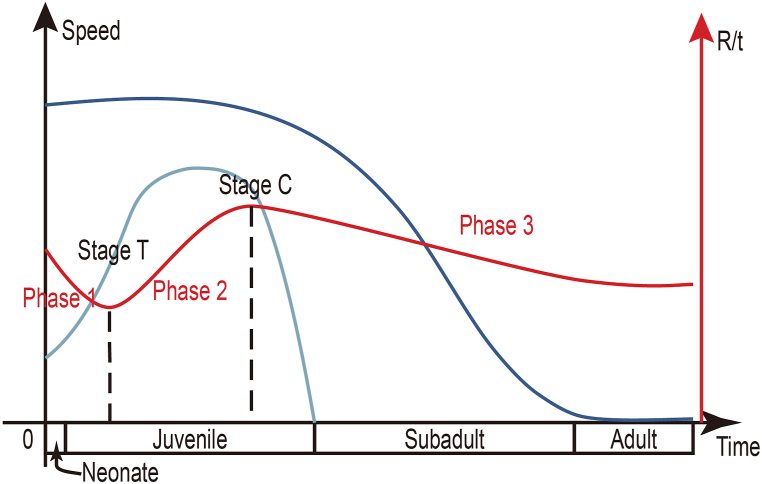
Schematic diagram showing the changes of the R/t ratio during the ontogeny of pterosaurs (red curve). The dark and light blue curves represent the expansion of the cortex and medullary cavity, respectively. See text for details.

Many juveniles show low R/t ratios. There are four pterosaur specimens with low R/t ratios of less than 3, excluding non-pterodactyloids, archaeopterodactyloids, and dsungaripterids (Table S1). Two of them are juvenile specimens of *Sinopterus* (IVPP V 13363 & V 14430) with unfused scapula and coracoid [65,66]. The ontogenetic stages of the others are unknown. The humerus of *Pterodaustro* had an extremely low R/t ratio (Table S1), although the bone wall thickness of *Pterodaustro* was considered extremely thin [46]. It is the smallest individual in a series of limb bones, only 13 % of the largest, and it is undoubtful that this humerus represents a juvenile. Another low R/t ratio not included in Table S1 was an azhdarchoid, also a juvenile individual [61]. Although Martin-Silverstone [18] thought body size rather than ontogenetic stages influence the relative thickness, the latter is preferred here because of the procedure of bone growth. In the neonates of basal archosaurs and some other reptiles, the cortex is thin, and the medullary cavity is quite large [67], which is also discovered in an embryo of *Hamipterus* [68]. Hence, it is safe to assume a similar condition in pterosaurs in general. According to the histological study of *Pterodaustro* and *Hamipterus* [45,68], the change in the relative thickness of the bones, or R/t ratios, can be divided into three phases (Fig. 6). In the first phase, starting from the neonate to stage T (slightly before the speed peak of medullary expansion), the cortex expands much faster than the medullary cavity, which makes the R/t ratio reach the first trough. The juveniles with low R/t ratios are in this phase. In the second phase, from stage T to stage C (slightly before reaching the subadult stage), expansion of the cavity becomes quite slow, while the expansion of the cortex remains fast, so the R/t ratio reaches the crest. The last phase is from stage C to the end of life. During this phase, the expansion of the cavity almost ceases, and the cortex continues growing. The R/t ratio would reach another trough if the pterosaur has a determinate growth or the ratio becomes the largest until death. Low R/t ratios of adult pterosaurs were obtained in this phase. Hence, the low R/t ratios should be treated cautiously because there are two troughs during ontogeny.

Relatively thick bone walls often suggest two possible lifestyles among extant and extinct vertebrates: burrowing or fossorial lifestyle [[69], [70], [71]] and diving or aquatic lifestyle [[72], [73], [74], [75]]. The bone wall thickness of more than 30 % of the diameter (R/t ratio smaller than 1.67) is one indication of not only fossorial but also aquatic lifestyles [71,72]. In our dataset, the R/t ratios of dsungaripterids and non-pterodactyloids do not reach the threshold; and six archaeopterodactyloids specimens, including *Pterodactylus*, *Germanodactylus*, and three ctenochasmatids, have R/t-ratios around this threshold (Table S1). An aquatic or semi-aquatic lifestyle is rather possible than a fossorial lifestyle for archaeopterodactyloids, because of their food (such as fish, hard-shelled invertebrates, and small aquatic creatures [76]) and the interdigital webbing in some taxa [[77], [78], [79]]. The thick bone walls of creatures in this lifestyle are generally interpreted to pertain to decreased buoyancy in water [73,75]. However, each of the six specimens has R/t ratios of no more than three limb bones, and some of them are far from the threshold. Hence, wall thicknesses of limited limb bones are not adequate to build connections with their possible lifestyles, and more information is needed.

### 5.3 Relationships of *Cratonopterus huabei*

The wing metacarpal of the new material is elongated, and it is confident to exclude its referral to non-pterodactyloids, which have much larger humerus/metacarpal-IV ratios (Table 1; [80]). The elongated mid-cervical vertebra with a low-ridged neural spine is a typical characteristic of the Archaeopterodactyloidea or the Azhdarchidae [22,23]. Regarding the shape of the deltopectoral crest of the humerus and the absence of the notarium as well as its medium size, the assignment of the Azhdarchidae should also be excluded. Compared to the main postcranial ratios of most archaeopterodactyloids and some other pterosaurs (Table 1), only ctenochasmatids, *Forfexopterus jeholensis*, *Elanodactylus prolatus*, and *Eosipterus yangi*, have all four ratios similar to those of the new material. Based on these ratios, it can be safely assigned to the Ctenochasmatidae.

**Table 1:** Measurements (in mm) and ratios of the forelimbs of pterosaurs. Compared with the new material, the ratios with percentage changes of less than 15 % are highlighted in bold. See institutional abbreviations in the Supplementary material.

	hu	mcIV	ph1d4	ph3d4	hu /mcIV	ph1d4 /mcIV	ph1d4 /hu	ph1d4 /ph3d4	notes
Non-pterodactyloids									
***Dorygnathus banthensis* UUPM R 156**	61	29	73	88	2.10	2.52	1.20	0.83	[81]
***Rhamphorhynchus longicaudus* TM 6924**	16.5	10	37	28.9	1.65	3.70	2.24	1.28	[82]
***Qinglongopterus guoi* D3080/3081**	17.8	9.1	31.7	29.7	1.96	3.48	1.78	**1.07**	[83]
***Jeholopterus ningchengensis* IVPP V 12705**	62	19^a^	93	60	3.26	4.89	**1.50**	1.55	[84]
***Darwinopterus robustodens* 41HIII-0309A**	50	30	65	75	1.67	2.17	**1.30**	**0.87**	[85]
***Douzhanopterus zhengi* STM 19**–**35**	48.49	31.72	53.26	53.19	1.53	**1.68**	1.10	**1.00**	[80]
Pterodactyloidea									
Ctenochasmatidae									
***Cratonopterus huabei* gen. et sp. nov. IVPP V 14395**	113^a^	102.81	159^a^	157.07	1.10	1.55	1.41	1.01	This paper
***Ctenochasma elegans* BSPG 1875.XIV.501**	15	16.5	21.3	15.5	0.91	1.29	**1.42**	1.37	[86]
***Ctenochasma elegans* BSPG 1935.I.24**	38.5	52^a^	66	–	0.74	1.27	1.71	–	[86]
***Aurorazhadarcho micronyx* ELTE V 256**	20.7	26.1	33.7	21.8	0.79	1.29	1.63	1.55	[87]
***Aurorazhadarcho micronyx* BSPG 1911.I.31**	25	35	46	19.5	0.71	1.31	1.84	2.36	[82]
***Pterodaustro guinazui* PVL 3860**	80	78	116	85	**1.03**	**1.49**	**1.45**	1.36	[82]
***Forfexopterus jeholensis* HM V20**	118.3	191.1	217.6	135.4	0.62	1.14	1.84	1.61	[26]
***Forfexopterus jeholensis* SDUST-V 1003**	103	110^a^	147.6	131.9	**0.94**	**1.34**	**1.43**	**1.12**	[88]
***Huanhepterus quingyangensis* IVPP V 9070**	145^a^	–	360	–	–	–	2.48	–	[38]
***Gladocephaloideus jingangshanensis* JPM-2014**–**004**	30	32.4^a^	45.5	37.5	0.93	**1.40**	**1.52**	1.21	[89]
***Gegepterus changae* IVPP V 11981**	–	52.7^a^	70.8^a^	–	–	**1.34**	–	–	[29]
***Elanodactylus prolatus* GMC V2330**	147.1	126.5^a^	208.9	212.1	**1.16**	**1.65**	**1.42**	**0.98**	[30]
***Elanodactylus prolatus* LPM-R00078**	–	105	148	152	–	**1.41**	–	**0.97**	[27]
***Beipiaoopterus chenianus* BPM 0002**	68	75	–	85	0.91	–	–	–	[90]
***Eosipterus yangi* GMC V2117**	–	73^a^	96	80	–	**1.32**	–	1.20	[91]
***Eosipterus yangi* D2514**	47	42	63	55	**1.12**	**1.50**	**1.34**	**1.15**	[92]
Non-ctenochasmatids									
***Germanodactylus cristatus* BSPG 1892.IV.1**	56	66	84	65.5	0.85	1.27	**1.50**	1.28	[82]
***Germanodactylus rhamphastinus* BSPG AS.I.745**	60	70	90^a^	72	0.86	1.29	**1.50**	1.25	[82]
***Pterodactylus antiquus* BSPG AS.I.739**	31.5	35	48.5	37	0.90	**1.39**	**1.54**	1.31	[82]
***Pterodactylus antiquus* BSPG AS.XIX.3**	28.5	30.5	40	31.4	0.93	1.31	**1.40**	1.27	[82]
***Ardeadactylus longicollum* SMNS 56603**	78	130	160	77.5	0.60	1.23	2.05	2.06	[82]
***Cycnorhamphus suevicus* GPIT 80**	65.5	108	141	85	0.61	1.31	2.15	1.66	[82]
***Sinopterus dongi* IVPP V 13363**	59	95	121	63	0.62	1.27	2.05	1.92	[65]
***Istiodactylus sinensis* GMC V2329**	133.5	162.3^a^	273.1^a^	195.4	0.82	1.68	2.05	1.40	[93]
***Noripterus complicidens* IVPP RV 73001**	77	140	165	–	0.55	1.18	2.14	–	[34]
***Jidapterus edentus* RCPS-030366CY**	78.6	145.3	185.4	74.6	0.54	1.28	2.36	2.49	[94]
***Santanadactylus pricei* AMNH 22555**	170	172	372	252	**0.99**	2.16	2.19	1.48	[95]
***Pteranodon* NHMUK 2959**	246	549	577	309	0.45	1.05	2.35	1.87	[35]
***Zhejiangopterus linhaiensis* ZMNH M1323**	137	336	322	–	0.41	0.96	2.35	–	[96]

a Estimated value.

A large pneumatic foramen located at the ventral surface of the proximal end of the first wing phalanx is present in the Ornithocheiroidea, such as *Pteranodon*, *Anhanguera*, *Hamipterus* [32,33,35], but absent in known archaeopterodactyloids, such as *Pterodactylus*, *Germanodactylus rhamphastinus*, *Ardeadactylus*, *Forfexopterus* [26,28], which are confirmed by the recent phylogenetic analysis [24]. Hence, the presence of this large pneumatic foramen can be considered one of the autapomorphies of this new taxon. Additionally, the coracoid has an expansion at its contact with the scapula, as in *Gegepterus*, *Elanodactylus*, and *Forfexopterus* [26,29,30]. This expansion is absent in the new taxon (Fig. 1), which is another autapomorphy.

Information regarding the sterna in the Ctenochasmatidae is limited. The cristospines of *Elanodactylus* and *Forfexopterus* [26,27] are longer than that of *Cratonopterus*. The only known complete sternal plate of a ctenochasmatid was fan-shaped in *Forfexopterus* [26]. Other ctenochasmatid sternal plates are incomplete. The holotype of *Elanodactylus prolatus* preserved its lateral margins similar to that of *Cratonopterus*, and the anterior margin of the former is slightly more curved [30]. The referred specimen of *E. prolatus* had a quite short anterior margin relative to its posterior margin or whole length [27]. *Huanhepterus* also had a short anterior margin, which was misinterpreted as the posterior margin previously [38]. The preserved part of the sternal plate of *Gegepterus* was quite similar to that of *Cratonopterus* [29], and the only difference was that the posterior margin of the former was straighter than that of the latter.

The four main postcranial ratios of *Cratonopterus* can be used to distinguish it from most ctenochasmatids, except for *E. prolatus* (Table 1). Notwithstanding these ratios, the distinct tubercle on the proximal margin of the humerus between the deltopectoral crest and the head is absent in the new material [30]. Hence, *Cratonopterus* differs from other ctenochasmatids based on the combination of these characteristics.

Only one pterosaur specimen (SDUST-V1006) was reported from the Huajiying Formation and has been assigned to the Ornithocheiroidea [15]. Unfortunately, the overlaps between the previous specimen and *Cratonopterus* are some parts of wing phalanges, providing limited information for taxonomic study. The pedal configuration of SDUST-V1006 is divergent from that of ctenochasmatids, thus these two specimens represent different taxa. The Huajiying Formation, the deposits of the early stage of the Jehol Biota (135.4–128.7 Ma), crops out in the Sichakou-Senjitu basins of Fengning, Hebei Province [6]. Based on the two complete bony fish and conchostracans, probably *Lycoptera* and *Eosestheria*, *Cratonopterus* came from the upper part of the Huajiying Formation. This is consistent with the horizon of SDUST-V1006, showing the pterosaur diversity at the early stage of the Jehol Biota. Although the exact age is unknown, these two pterosaur specimens are at least 4 million year older than the earliest known pterosaurs from the Yixian Formation.

Ctenochasmatid pterosaurs have been reported from all over the world, especially in northeastern China with high diversity [9,97]. Except for the possible ctenochasmatid tooth from the Middle Jurassic [98], the earliest known ctenochasmatid is *Liaodactylus*, which was from the Late Jurassic Yanliao Biota in China [97]. Before the discovery of the new material, the second earliest ctenochasmatids in China included multiple taxa from the lower part of the Yixian Formation (125.4 Ma) [9,16,17], with a time gap of approximately 35 million years from the earliest one. Little is known about the ctenochasmatids in western Liaoning during this period. The discovery reduces the gap in the evolution of ctenochasmatids in western Liaoning, indicating that this clade of pterosaurs probably existed and lasted for a long time in this region.

## Data availability statement

Data included in article/supp. material/referenced in article.

## CRediT authorship contribution statement

**Shunxing Jiang:** Conceptualization, Data curation, Formal analysis, Funding acquisition, Investigation, Methodology, Visualization, Writing – original draft, Writing – review & editing. **Junyi Song:** Data curation, Investigation, Writing – review & editing. **Xinjun Zhang:** Data curation, Investigation, Writing – review & editing. **Xin Cheng:** Data curation, Investigation, Writing – review & editing. **Xiaolin Wang:** Conceptualization, Funding acquisition, Resources, Software, Supervision, Writing – review & editing.

## Declaration of competing interest

The authors declare that they have no known competing financial interests or personal relationships that could have appeared to influence the work reported in this paper.
