# Supplementary material for: A new pterosaur from the early stage of the Jehol biota in China, with a study on the relative thickness of bone walls
Source: Heliyon. 2023 Nov 17;9(12):e22370. doi: 10.1016/j.heliyon.2023.e22370 (PMC10709016; doi:10.1016/j.heliyon.2023.e22370)
Supplement: Multimedia component 2 [file mmc2.docx]

**A new pterosaur from the early stage of the Jehol Biota in China, with a study on the relative thickness of bone walls**

Shunxing Jiang^1^*, Junyi Song^1,2^, Xinjun Zhang^3,1,2^, Xin Cheng^4^, Xiaolin Wang^1,2^*

^1^Key Laboratory of Vertebrate Evolution and Human Origins, Institute of Vertebrate Paleontology and Paleoanthropology, Chinese Academy of Sciences (CAS), Beijing, 100044, China

^2^College of Earth and Planetary Sciences, University of Chinese Academy of Sciences, Beijing, 100049, China

^3^College of Paleontology, Shenyang Normal University, Shenyang, 110034, China

^4^College of Earth Sciences, Jilin University, Changchun, 130061, China

*Correspondence to: Shunxing Jiang (jiangshunxing@ivpp.ac.cn) or Xiaolin Wang (wangxiaolin@ivpp.ac.cn)

Institutional abbreviations.

Figs S1, S2.

Supplementary references.

**Institutional abbreviations.**

41HIII, Henan Natural History Museum, Zhengzhou, China; AMNH, American Museum of Natural History, New York, USA; BPM, Beipiao Museum of Liaoning Province, China; BSPG, Bayerische Staatssammlung für Paläontologie und Geologie, Munich, Germany; CM, Carnegie Museum of Natural History, Pittsburgh, USA; CPE, Centro Paleontológico de Enciso, Spain; D, Dalian Natural History Museum, China; ELTE, Eötvös University, Budapest, Hungary; GMC, Geological Museum of China, Beijing, China; GPIT, Geologisch-Paläontologisches Institut, Universitat Tübingen, Germany; HM, Hami Museum, Hami, China; IBP, Steinmann Institut für Geologie, Mineralogie und Paläontologie, Bonn, Germany; IVPP, Institute of Vertebrate Paleontology and Paleoanthropology, Chinese Academy of Sciences, Beijing, China; JPM, Jinzhou Paleontological Museum, China; LACM, Los Angeles County Museum, Los Angeles, USA; LPM, Liaoning Paleontological Museum, Shenyang Normal University, China; MHN-UNSL-GEO, Museo de Historia Natural de la Universidad Nacional de San Luis, Geología, Argentina; MN, Museu Nacional, Rio de Janeiro, Brazil; MPSC, Museu de Paleontologia de Santana do Cariri, Brazil; NHMUK, Natural History Museum, London, UK; NMS, National Museums Scotland, Edinburgh, UK; NSM-PV, Division of Vertebrate Paleontology, National Science Museum, Tokyo, Japan; PVL, Instituto Miguel Lillo, Universidad Nacional de Tucumán, San Miguel de Tucumán, Argentina; RAM, Raymond M. Alf Museum of Paleontology, Claremont, USA; RBCM, Royal British Columbia Museum, Victoria, Canada; RCPS, Research Center of Palaeontology & Stratigraphy of Jilin University, Changchun, China; SDUST, Shandong University of Science and Technology, Qingdao, China; SGP, Sino-German Project Collection, currently held at GPIT, Tübingen, Germany; SMNK, Staatliches Museum für Naturkunde, Karlsruhe, Germany; SMNS, Staatliches Museum für Naturkunde Stuttgart, Germany; STM, Shangdong Tianyu Museum of Nature, Pingyi, China; TM, Teylers Museum, Haarlem, Netherlands; TMP, Royal Tyrrell Museum of Palaeontology, Drumheller, Canada; UUPM, Palaeontological Museum, University of Uppsala, Sweden; UP, University of Portsmouth, Portsmouth, UK; ZMNH Zhejiang Museum of Natural History, Hangzhou, China.


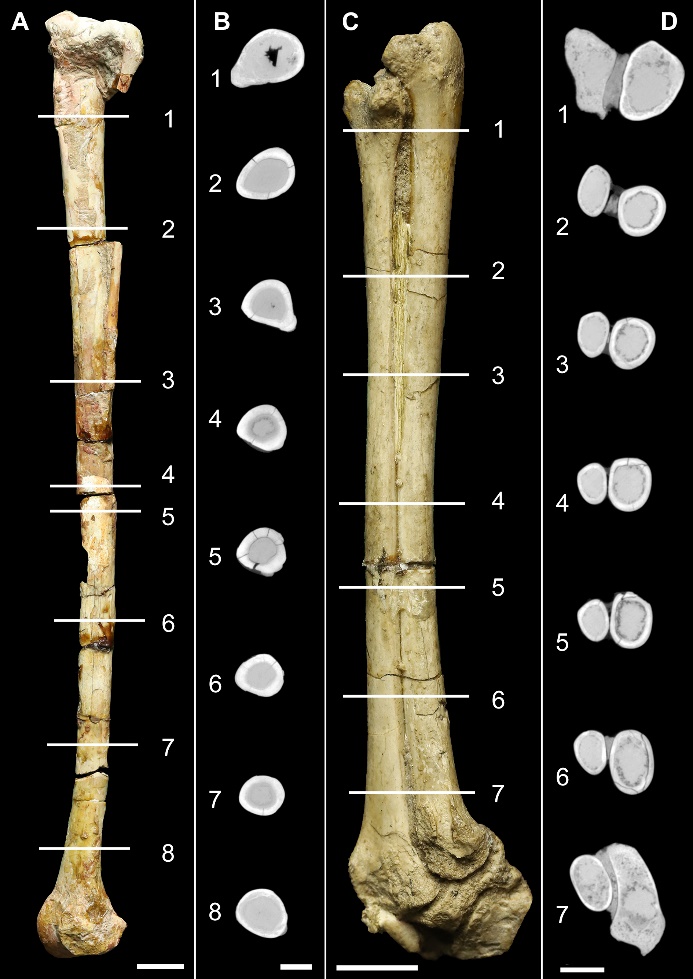


**Fig. S1.** Cross-sections of *Dsungaripterus* and *Noripterus* using CT scanning. A, tibia and fibula of *Dsungaripterus* *weii* (IVPP field No. 64041). B, cross-setions of the tibia and fibule in A. C, ulna and radius of *Noripterus complicidens* (IVPP RV 73001). D, cross-sections of the ulna and radius in C. Scale bars, 10 mm in A and C; 5 mm in B and D.

**
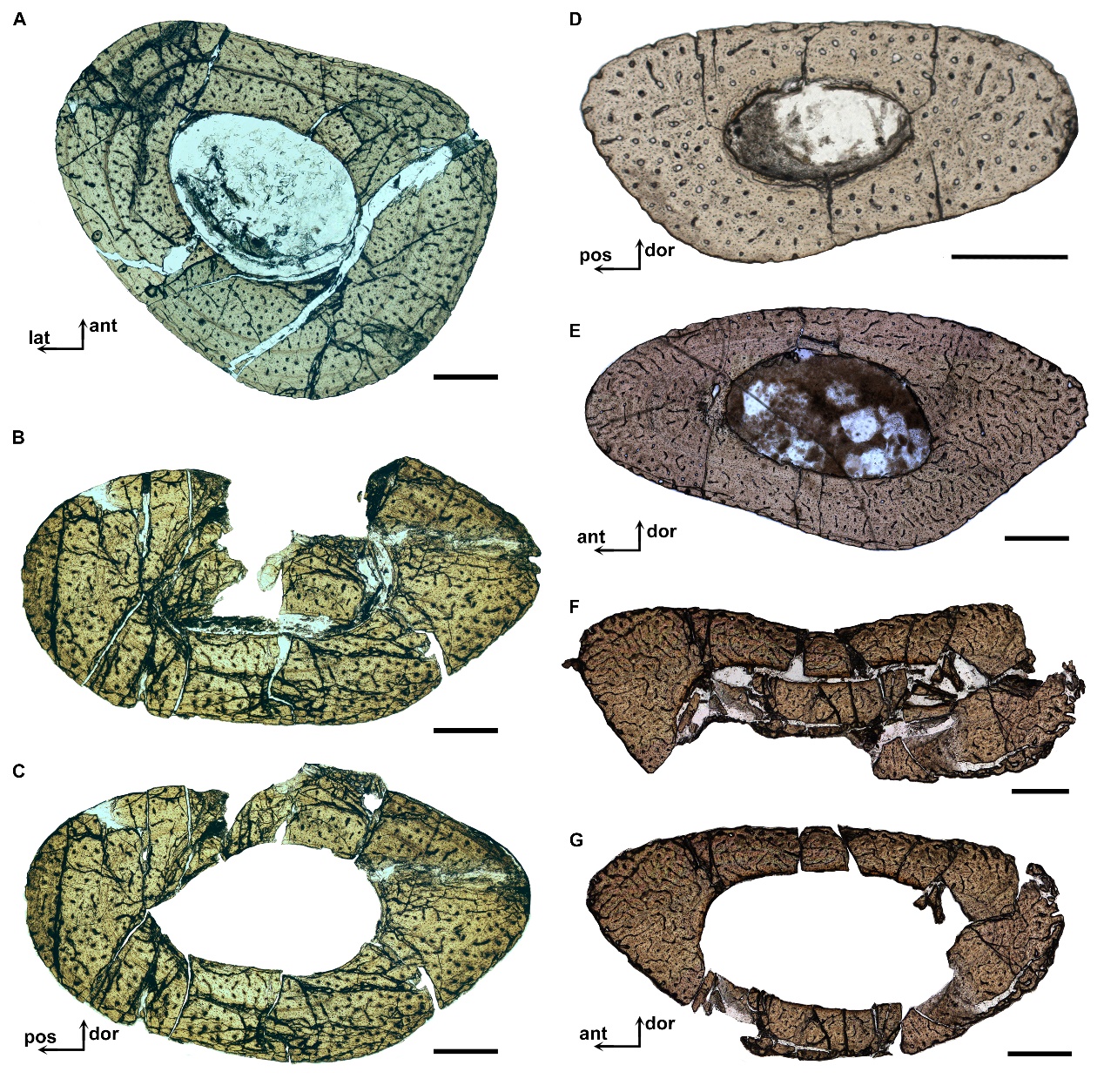
**

**Fig. S2.** Thin sections of non-dsungaripteroids with relatively thick bone walls. A, tibia of the ctenochasmatid (IVPP V 12693). B, second wing phalanx of the ctenochasmatid (IVPP V 12693). C, reconstruction of B. D, third wing phalanx of *Gegepterus changae* (IVPP V 11981). E, second wing phalanx of *Sinopterus dongi* (IVPP V 14430). F, second wing phalanx of *Sinopterus dongi* (IVPP V 13363). G, reconstruction of F. Abbrevieations: ant, anterior; dor, dorsal; lat lateral; pos, posterior. Scale bars, 500 μm.

**Supplementary references**

[S1] P. Wellnhofer, Die Rhamphorhynchoidea (Pterosauria) der Oberjura-Plattenkalke süddeutschlands Teil I, Palaeontogr Abt A. 148 (1-3) (1975) 1-33.

[S2] E. Prondvai, A. Ősi, Potential for intracranial movements in pterosaurs, Anat Rec. 294 (5) (2011) 813-830.

[S3] M. O'Sullivan, D.M. Martill, D. Groocock, A pterosaur humerus and scapulocoracoid from the Jurassic Whitby Mudstone Formation, and the evolution of large body size in early pterosaurs, P Geologist Assoc. 124 (6) (2013) 973-981.

[S4] E. Prondvai, K. Stein, A. Ősi, M. Sander, Life history of *Rhamphorhynchus* inferred from bone histology and the diversity of pterosaurian growth strategies, PLoS ONE. 7 (2) (2012) e31392.

[S5] B. Andres, J.M. Clark, X. Xu, A New Rhamphorhynchid Pterosaur from the Upper Jurassic of Xinjiang, China, and the Phylogenetic Relationships of Basal Pterosaurs, J Vert Paleont. 30 (1) (2010) 163-187, https://doi.org/10.1080/02724630903409220.

[S6] N. Jagielska, M. O’Sullivan, G.F. Funston, I.B. Butler, T.J. Challands, N.D.L. Clark, N.C. Fraser, A. Penny, D.A. Ross, M. Wilkinson, S.L. Brusatte, A skeleton from the Middle Jurassic of Scotland illuminates an earlier origin of large pterosaurs, Curr Biol. (2022) 1-8, https://doi.org/10.1016/j.cub.2022.01.073.

[S7] K. Padian, J.R. Horner, A.J. de Ricqlès, Growth in small dinosaurs and pterosaurs: the evolution of archosaurian growth strategies, J Vert Paleont. 24 (3) (2004) 555-571.

[S8] D.W.E. Hone, M.B. Habib, M.C. Lamanna, An annotated and illustrated catalogue of Solnhofen (Upper Jurassic, Germany) pterosaur specimens at Carnegie Museum of Natural History, Ann Carnegie Mus. 82 (2) (2013) 165-191.

[S9] S.C. Bennett, Juvenile specimens of the pterosaur *Germanodactylus cristatus*, with a review of the genus, J Vert Paleont. 26 (4) (2006) 872-878.

[S10] T. Rodrigues, A.W.A. Kellner, O. Rauhut, A new specimen of the archaeopterodactyloid *Germanodactylus ramphastinus*, Acta Geosci Sin. 31 (Suppl 1) (2010) 57-58.

[S11] X. Pereda‐Suberbiola, F. Knoll, J.I. Ruiz‐Ome aca, J. Company, F. Torcida Fernández-Baldor, Reassessment of *Prejanopterus curvirostris*, a basal pterodactyloid pterosaur from the Early Cretaceous of Spain, Acta Geol Sin. 86 (6) (2012) 1389-1401.

[S12] A. Oei, N. Carroll, A.A. Farke, An azhdarchid pterosaur from the Kaiparowits Formation of southern Utah, PaleoBios. 35 (Suppl) (2018) 22-22.

[S13] E.G. Martin-Silverstone, M.P. Witton, V.M. Arbour, P.J. Currie, A small azhdarchoid pterosaur from the latest Cretaceous, the age of flying giants, Roy Soc Open Sci. 3 (8) (2016) 160333, https://doi.org/10.1098/rsos.160333.

[S14] D.W.E. Hone, M.B. Habib, F. Therrien, *Cryodrakon boreas*, gen. et sp. nov., a Late Cretaceous Canadian azhdarchid pterosaur, J Vert Paleont. 39 (3) (2019), https://doi.org/10.1080/02724634.2019.1649681.

[S15] M.P. Witton, D.M. Martill, M. Green, On pterodactyloid diversity in the British Wealden (Lower Cretaceous) and a reappraisal of "*Palaeornis*" *cliftii* Mantell, 1844, Cretaceous Res. 30 (3) (2009) 676-686, https://doi.org/10.1016/j.cretres.2008.12.004.

[S16] E. Buffetaut, D. Grigorescu, Z. Csiki, A new giant pterosaur with a robust skull from the latest Cretaceous of Romania, Naturwissenschaften. 89 (4) (2002) 180-184, https://doi.org/10.1007/s00114-002-0307-1.

[S17] K. Padian, A.J. de Ricqlès, J.R. Horner, Bone histology determines identification of a new fossil taxon of pterosaur (Reptilia: Archosauria), C R Acad Sci Paris, Sér IIA. 320 (1995) 77-84.

[S18] K. Eck, R.A. Elgin, E. Frey, On the osteology of *Tapejara wellnhoferi* KELLNER 1989 and the first occurrence of a multiple specimen assemblage from the Santana Formation, Araripe Basin, NE-Brazil, Swiss J Palaeontol. 130 (2) (2011) 277-296, https://doi.org/10.1007/s13358-011-0024-5.

[S19] A.W.A. Kellner, Comments on the Pteranodontidae (Pterosauria, Pterodactyloidea) with the description of two new species, An Acad Bras Cienc. 82 (4) (2010) 1063-1084.

[S20] R.W. Hooley, On the skeleton of *Ornithodesmus latidens*; an ornithosaur from the Wealden Shales of Atherfield (Isle of Wight), Quart Journ Geol Soc. 69 (1-4) (1913) 372-422, https://doi.org/10.1144/gsl.jgs.1913.069.01-04.23.

[S21] M.P. Witton, New insights into the skull of *Istiodactylus latidens* (Ornithocheiroidea, Pterodactyloidea), PLoS ONE. 7 (3) (2012) e33170.

[S22] A.J. Veldmeijer, Preliminary description of a skull and wing of a Brazilian Cretaceous (Santana Formation; Aptian–Albian) pterosaur (Pterodactyloidea) in the collection of the AMNH, PalArch’s J Vertebr Palaeontol. (2003) 1-14.

[S23] R.A. Elgin, Palaeobiology, Morphology, and Flight Characteristics of Pterodactyloid Pterosaurs, Doctoral dissertation, University of Heidelberg, 2014, pp. 1-273.

[S24] P. Wellnhofer, Neue Pterosaurier aus der Santana-Formation (Apt) der Chapada do Araripe, Brasilien, Palaeontogr Abt A. 187 (4-6) (1985) 105-182.

[S25] A.J. Veldmeijer, Pterosaurs from the Lower Cretaceous of Brazil in the Stuttgart collection, Stuttgarter Beitr Naturk, Ser. B. 327 (2002) 1-27.

[S26] L.H.S. Eleutério, R.A.M. Bantim, F.J. Lima, R.C.L.P. Andrade, A.Á.F. Saraiva, A.W.A. Kellner, J.M. Sayão, Biomechanical and physiological influences on the osteohistological deposition of Anhangueria (Pterosauria, Pterodactyloidea), Rev Bras Paleontolog. 18 (3) (2015) 403-412.

[S27] B.C. Vila Nova, A.Á.F. Saraiva, J.K.R. Moreira, J.M. Sayão, Controlled excavations in the Romualdo Formation Lagerstätte (Araripe Basin, Brazil) and pterosaur diversity: remarks based on new findings, Palaios. 26 (3) (2011) 173-179, https://doi.org/10.2110/palo.2010.p10-072r.
